# Musculoskeletal characteristics of the lower limbs of students at a vocational ballet institution – retrospective analysis of cross-sectional data

**DOI:** 10.1186/s12891-025-08692-y

**Published:** 2025-05-06

**Authors:** Tobias Almasi, Elisabeth Exner-Grave, Daniela Ohlendorf, David A. Groneberg, Eileen M. Wanke

**Affiliations:** 1 Institute of Environmental, Social and Occupational Medicine, Goethe University, Frankfurt/Main, Germany; 2 Section of Social Affairs, District of Soest, Department of Youth, Education and Social affairs, Soest, Germany; 3 Institute of Occupational-, Social- and Environmental Medicine, Section Performing Arts Medicine, Goethe University, Theodor-Stern-Kai 7, 60590 Frankfurt, Germany

**Keywords:** Vocational students, Eligibility, Lower extremity, Muskuloskeletal characteristics, Classical dance, Ballet

## Abstract

**Background:**

The existing literature on pre-professional ballet students mostly reports on injury patterns, eating disorders, hormonal paramenters or other singular items whereas an overall description of anthropometric and orthopedic values of this particular study population can rarely be found. The aim of the present article was to anthropometrically and orthopedically describe the lower extremity of the typical vocational ballet student, establish a database and enable future comparisons.

**Methods:**

In this study, *n* = 606 students (414 female, 192 male) of a governmental institution for vocational ballet in Germany, between the ages of 5 and 22 (Mean ±SD: 13.9 ±3.5) were examined by an experienced orthopedist and dance physician.

**Results:**

The average passive external rotation of the hip (ER) was 60° (±7.5) and 43.5° (±11.3) for the internal rotation (IR). The calculated range of motion (ROM) was 103.3° (±10.4). The mean tibial torsion (TT) was 22.4° (±6.3). The average calculated turnout (ER + TT) was 83.3° (±9.9) per leg. The passive dorsiflexion (DF) of the ankle was 23.6° (±4.7) and 70.1° (±7.5) for plantarflexion (PF). 52.5% of the examinees showed a splay foot. The passive DF of the metatarsophalangeal (MTP) joint was 88.9° (±6.3) and 62.3° (±5.2) for PF. The Pearson’s correlation coefficient for age was *r* =.237/0.302 (right/left) with hip ER, *r*= -.634/0.537 (right/left) with hip IR, *r* =.11 (both sides) with ankle PF and *r* = −.0.253/-0.264 (right/left) with MTP joint DF of the big toe.

**Conclusions:**

The typical vocational ballet student of our study showed a large ROM for hip ER, ankle PF and MTP joint DF of the big toe. Students were more flexible than examinees of previous studies and often achieved ballet-specific ideal values. Hence, it is very likely that a ballet-specific selection favours the generally large ROMs found in the present study. The data collected over the long period of twenty years can be seen as a limitation of the present study, as students selected at the beginning of that period might be very different to selected students at the end it. Furthermore, this study measured passive joint range of motion only.

## Background

While the ballet pedagogy of classical ballet dates back to 19th century developments, strict selection and the systematization of long training beginning in childhood have led to enormous technical and stylistic development [1]. Even though technical fundamentals have remained unchanged, the demand for virtuosity, acrobatics, precision, speed and versatility in terms of mastering different styles has greatly increased in the 21st century [2–4]. In addition to artistic-aesthetic requirements, psychological and physical stresses, comparable to those experienced in competitive sports, have an effect on the growing ballet student [5]. Furthermore, there are hardly any aids that facilitate the work process and protect the dancer’s body. The stresses act directly on the body, which, for this profession, makes an unrestricted and uncompromising suitability of the body all the more important [5–7].

With limited suitability there are increased risks of acute injuries, incorrect loading and overuse damage. Main sites of injury and damage in ballet students are joint and ligament structures of the lower extremities, resulting in tendinopathies, stress fractures, or degeneration of cartilage and bone. Less common are injuries to the upper extremities [5, 8–12].

So what characteristics does the suitable body for professional classical ballet provide? The theoretic ideal of the physical characteristics for classical ballet are to be found in classical ballet literature. And even though extensive studies are sparce,

the physical characteristics of professional ballet dancers are well described in dance medicine literature [6, 13, 14]. For example, above-average flexibility is a characteristic of professional ballet dancers, as it is in acrobatic arts and gymnastics [6]. Furthermore, the external rotation of the hips plays a particularly important role in ballet, since all movements in classical ballet are based on outwardly rotated legs (turnout) [2]. However, all this data refers to the grown and developed body of the adult professional dancer but little is known about the growing body of the vocational ballet student [14–18].

The aim of the present study therefore was to examine students of a vocational school for classical ballet for anthropometric and orthopedic characteristics in order to provide a broad and up-to-date database that would describe the typical vocational ballet student. The results of this article are part of a dissertation to attain the german medical doctor’s degree.

## Methods

The present study is a retrospective, exploratory analysis of cross-sectional data collected between 1998 and 2017. The medical doctor perfoming the examinations is a senior orthopedist and accredited dance medicine specialist and remained the same over the whole period of data collection. *N* = 606 students of a vocational ballet institution (414 females, 192 males) were interviewed and examined for sociodemographic, anthropometric and functional characteristics. They were of an average age of 13.9 years (SD ±3.5, range: 5–22) and of high cross-cultural diversity (49 countries, 5 continents). Table 1 shows the respective n per age group and sex of the study population. We were unable to calculate the age for two female and one male examine because of documentation errors. Students were in the ongoing admission process for ballet school (pre-school, grades 1–6), vocational school/state ballet academy, or had already been admitted to a program of the state training institution. Students were grouped by age into younger and older students following the structure of the schools educational program into pre-school (age < 10 years) and professional branch (age ≥ 10 years) to test for possible significant differences between age groups in general and between preparatory and vocational students in specific. Significant differences between those age groups we tested for Pearson’s correlation. Furthermore we grouped into children (< 10), preadolescence [10–18] and adults (≥ 18 years) for more detailed analysis of possible differences between age groups as a mere display of data. The medical examination was part of the admission process to enter the school. In general, students were seen once. Only a few students with very small or large range of motions, acute injuries or inflammations, for example, received a follow up. Follow up examinations focused on the development of these findings only. The data from those longitudinal assessments were not included in this study.

**Table 1:** N by age group and sex (female/male: *n* = 412/191, missing: 2/1)

Age	5	6	7	8	9	10	11	12	13	14	15	16	17	18	19	20	22
f – m	2 – 0	3 – 0	27 – 2	30 – 10	30 – 8	40 – 5	30 – 10	15 – 9	21 – 20	45 – 12	42 – 25	16 – 27	17 –27	18 – 24	5 – 7	2 – 4	0 – 1

age in years, f = female, m = male

Examined characteristics were accordingly assessed as quantitative or qualitative expressions. Data collection and documentation was carried out using a laptop, examination table, measuring tape and Jules Rippstein’s [19] Plurimeter system: Pluritor C to measure external and internal rotation (ER/IR) of the hip in prone position, hip extended, knee flexed 90° and pelvis fixed to the table with the free hand; Pluritor T to measure tibial torsion (TT) in quadruped stance, feet protruding over the edge of the table, with the two tips of the measuring device touching the malleoli dorsally; Pluri-Ped to measure ankle range of motion (ROM) in supine, legs stretched and the plate firmly attached to the sole of the foot to avoid movement of other joints of the foot [6, 13, 19]. Leg axis, intercondylar distance, rearfoot axis (RFA), foot arch and big toe axis were measured or assessed in stance; leg length, foot shape, extension of the knee joint and metatarsophalangeal joint of the big toe (MTP) were measured or assessed in supine. The Plurimeter system allows an easy but precise and acurate measurement of angles whereas the professional experience of the same senior orthopedist and dance physician guarantees a relatively objective assessment of qualitative items.

Before the study began, a positive ethical vote was obtained from the ethics committee of the Goethe University Frankfurt am Main, Germany (No.: 2021-63, dated: 16.2.2021). Data preparation and analysis were performed by Microsoft Excel (Version 16.64) and IBM SPSS Statistics (Version 26 for Mac). We report mean, standard deviation and frequency in percent. We performed normality analysis for each characteristic category. Significance between age groups was tested by two-sided T test (normal distribution), Mann-Whitney U test (non-normal distribution) and Chi-Squared test (non numerical characteristics), with the significance level being set at 5.0%. For significant differences Pearson’s correlation was then used to evaluate the association between age and other variables. We interpreted coefficient values (*r*) from 0 to ±0.3 as negligible, ±0.31 to ±0.5 as low, ±0.51 to ±0.7 as moderate, ±0.71 to ±0.9 as high and values above as very high [20]. Missing data in each characteristic category resulted in different “n”s. The respective n are given in Tables 2 and in the other tables after each category. This article often cites two German speaking physicians whose references are to be ranked as expert opinion in dance medicine and orthopedics [6, 13, 21].

**Table 2:** Surveyed characteristics with respective N

Type	Body region	Characteristic (unit)	*n*
Anthropometry	Leg	Axis* Length (cm) ICD (TF) Tibial torsion (°)	588 584 552 392
	Foot	Rearfoot axis* Instep* Longitudinal arch* Transverse arch* Big toe axis* Shape*	591 86 551 577 593 585
Functionality	Hip Knee Ankle Tarsus MTPj	ER/IR (°) Extension (°) Extension/Flexion (°) Mobility* Extension (°) Flexion (°)	604 574 534 164 576 575

*qualitativ, ICD = Intercondylar distance, TF = transverse finger, ER/IR = external/internal rotation, MTP = Metatarsophalangeal joint of the big toe

## Results

### Legs and Hips - Inspection

Table 3 displays metric variables of the examination of legs and hips. The leg axis was straight in 92.0% of the ballet students. This was evident at intercondylar distances from 0 to 5 transverse fingers. From a distance of four transverse fingers, however, genua vara (4.8%) predominated in the visual impression. Genua valga were described in 3.2% of those examined. Legs of equal length were described for 96.1% of the subjects, while 3.9% showed a relevant difference in leg length (≥ 1.5 cm). Younger subjects showed significantly larger values for TT (age < 10: 16.9° ±6.1°) than older subjects (age ≥ 10: 23.5° ±5.7°; t(390)= -8.45, *p* <.001).

**Table 3:** Legs and hips

Characteristic (unit)	Mean ±SD (Range)	*n*
Leg length (cm)	85.4 ±10.5 (59.0-110.0)	584
ICD (TF)	0.8 ±1.2 (0–7)	552
ER (°)	60.0 ±7.5 (35–80)	604
IR (°)	43.5 ±11.3 (10–72)	604
ROM (°)	103.4 ±10.4 (72.5-138.5)	604
TT (°)	22.4 ±6.3 (3–40)	392

ICD = Intercondylar distance, TF = Transverse finger, ER/IR = External/Internal rotation of the hip, ROM = Range of Motion, TT = Tibial torsion

### Functional examination

83.7% of the ballet students showed a physiological extensibility of the knee joint (0–10°). Moderate (10–15°) and pronounced (> 15°) genua recurvata were documented for 12.4% and 2.6%, respectively. Genua recurvata were significantly more common among older participants (age < 10 vs. ≥ 10: 5.6/17.2%, χ² [1] = 9.281, *p* <.01) and here particularly among adolescents (10–14/14–18/≥18 years: 12.4/20.2/15.5%). In comparison to younger participants (age < 10: 56.1° ±6.7°) older subjects showed significantly greater hip ER (age ≥ 10: 60.8° ±6.8°; t(600)= -6.64, *p* <.001). On the other hand younger participants showed significantly greater values for hip IR (age < 10: 53.8° ±7.4°; age ≥ 10: 41.1° ±9.8°; t(210.65) = 15.30, *p* <.001). They also showed significantly larger hip ROM (age < 10: 109.9° ±8.5°) than older participants (age ≥ 10: 101.9° ±10.3°; t(193.28) = 8.64, *p* <.001). Table 4 demonstrates mean and SD of ER, IR and ROM of the hips across age groups. The correlation coefficient for ER and age (right/left) was *r* =.24/0.30 (*p* <.001) and *r*= -.63/-0.54 (*p* <.001) for IR and age.

**Table 4:** Hip mobility (*n* = 602), tibial torsion and turnout (*n* = 392) by age groups in degrees, (Mean ±SD)

Characteristic	10–14 years	14–18 years	≥ 18 years
Hip ER	59.0 ±7.2	61.8 ±6.5	60.7 ±6.3
Hip IR	47.7 ±7.9	38.7 ±9.1	35.8 ±9.3
Hip ROM (ER + IR)	107.7 ±9.4	100.5 ±9.8	96.5 ±10.1
TT	23.2 ±5.9	23.7 ±5.6	22.9 ±5.6
Turnout (ER + TT)	82.1 ±9.4	86.6 ±8.6	85.6 ±8.8

ER/IR = External/Internal rotation, ROM = Range of Motion, TT = Tibial torsion

### Turnout

The calculated average turnout (ER + TT) of those examined was 83.3° (±9.9°) per leg. Younger examinees showed significantly smaller turnout (age < 10: 74.7° ±9.5°) than older students (age ≥ 10: 85.1° ±9.0°; t(390)= -8.41, *p* <.001). Mean value was highest in the group of adolescents and adults (Table 4).

### Ankle, rearfoot and midfoot - Inspection

Table 5 displays frequencies of categorial characteristics of the foot examination. Valgus RFA was significantly more common among younger subjects (age < 10 vs. ≥ 10: 69.4/20.5%, χ² [1] = 103.33, *p* <.001). Prevalence was lowest among adults (10–14/14–18/≥18 years: 45.3/9.6/6.9%. A varus RFA was observed significantly more frequently among older examinees (age < 10 vs. ≥ 10: 2.7/15.0%, χ² [1] = 12.34, *p* <.001). Prevalence was highest among adolescents and adults (10–14/14–18/≥18 years: 5.3/19.6/19.0%). Flat feet were significantly more common among younger subjects (age < 10 vs. ≥ 10: 93.5/83.0%, χ² [1] = 7.52, *p* <.01). Prevalence was lowest among adolescents (10–14/14–18/≥18 years: 87.5/80.2/83.6%). Prevalence of a preserved longitudinal foot arch was significantly higher among older participants (age < 10 vs. ≥ 10: 6.5/14.7%, χ² [1] = 5.16, *p* =.02) and highest among adolescents (10–14/14–18/≥18 years: 11.8/16.9/12.7%). Hollow feet were first described at the age of 13 (10–14/14–18/≥18 years: 0.7/2.9/3.9%). Splayed feet occurred with a significantly higher prevalence among older students (age < 10 vs. ≥ 10: 33.6/57.1%, χ² [1] = 19.62, *p* <.001) and with highest prevalence among adolescents and adults (10–14/14–18/≥18 years: 49.0/61.2/58.6%). A splayfoot was found in 51.9% of those examined with pes planus, 42.5% of those with a preserved foot arch, and 30.0% of those with pes cavus.

**Table 5:** Examinations of the foot in percent

Characteristic		%	*n*
RFA	valgic straight varic	29.6 57.7 12.7	591
Instep	high normal low	39.5 19.8 40.7	86
Tarsus	flexible normal rigid	70.1 15.9 14.0	164
Arch (l)	lowered preserved elevated	84.9 13.3 1.8	551
Arch (t)	splayed not splayed	52.5 47.5	577

RFA = Rear foot axis, l/t = longitudinal/transverse

### Functional examination

Table 6 shows results for the examination of the ankle. Younger examinees showed significantly smaller plantarflexion (PF) of the ankle (age < 10: 67.6° ±5.7°) than older students (age ≥ 10: 70.7° ±7.8°; t(187.389)= -4.418, *p* <.001). The mean was highest among adolescents (Table 7). There was no significant difference in dorsiflexion (DF) of the ankle between younger (age < 10: 22.9° ±4.5°) and older students (age ≥ 10: 23.8° ±4.8°; t(531)= -1.624, *p* =.11) The mean was hightest among (pre)adolescents (Table 7). The correlation coefficient for PF and age (both sides) was *r* =.11 (*p* =.01).

**Table 6:** Passive ROM of ankle and MTP joint in degrees

Characteristic		Mean ±SD (Range)	*n*
ankle	dorsiflexion plantarflexion	23.6 ±4.7 (10–45) 70.1 ±7.5 (40–90)	534 534
MTP	dorsiflexion plantarflexion	88.9 ±6.3 (40–110) 62.3 ±5.2 (50–90)	576 575

ROM = Range of Motion, MTP = Metatarsophalangeal joint of the big toe

### Forefoot - Inspection

**Fig. 1:**
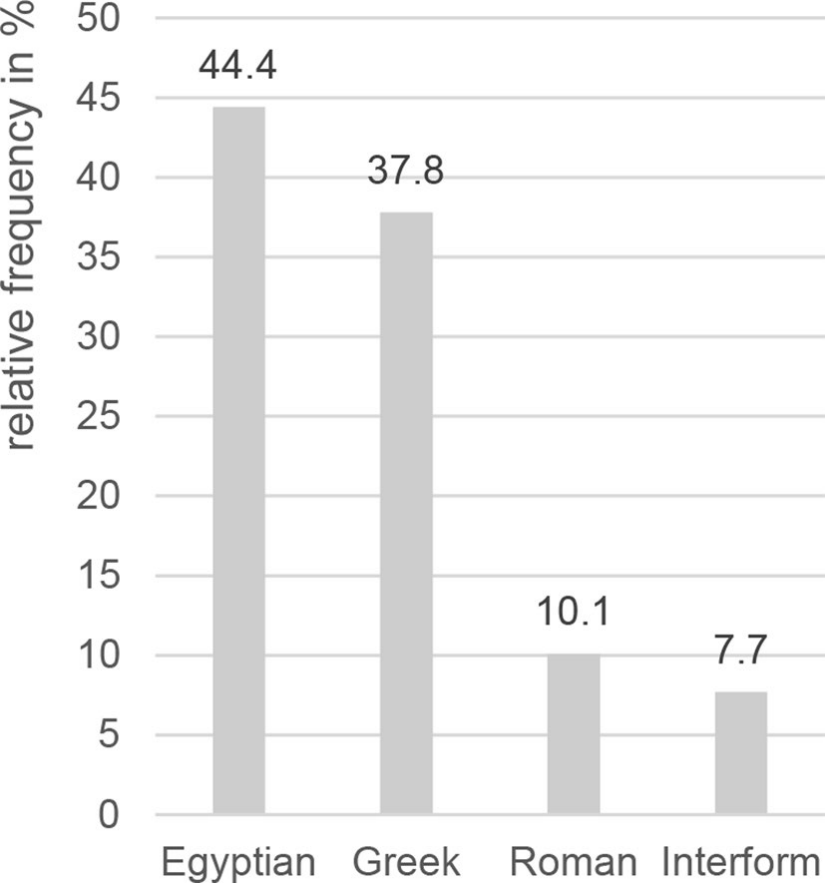
Foot shapes (*n* = 585)

The big toe axis was described as “straight” for 83.3% of those examined and as “valgus” for 16.7%. Prevalence of a valgus was highest among (pre)adolescents (< 10/10–14/14–18/≥18 years: 2.7/20.1/21.2/12.1%). Hallux valgus interphalangeus showed a prevalence of 9.1%. It was highest among (pre)adolescents (< 10/10–14/14–18/≥18 years: 2.7/11.4/10.6/8.6%). Egyptian and Greek foot shape was observed most frequently (Fig. 1). In 86.0% of cases with a Greek foot, there was a lengthened second metatarsal bone. In the remaining cases, the second toe but not the os metatarsale II was elongated.

**Table 7:** Passive ROM of ankle (*n* = 533) and MTP joint (*n* = 574) by age groups in degreees, (Mean ±SD)

Characteristic		10–14 years	14–18 years	≥ 18 years
Ankle	dorsifelxion plantarflexion	24.0 ±5.1 70.3 ±8.2	23.9 ±4.5 71.0 ±7.8	22.5 ±5.2 69.9 ±6.7
MTP	dorsiflexion plantarflexion	90.5 ±4.8 62.8 ±5.5	87.7 ±6.4 62.2 ±5.4	86.6 ±8.9 62.7 ±6.0

ROM = Range of Motion, MTP = Metatarsophalangeal joint of the big toe

### Functional examination

Younger participants showed significantly larger DF of the MTP joint (age < 10: 91.1° ±4.7°) than older subjects (age ≥ 10: 88.4° ±6.5°; U = 19734.50, Z= -4.22, *p* <.001) The mean was lowest among adults (Table 7). The correlation coefficient for DF of the MTP joint and age (right/left) was *r*= -.25/-0.26 (*p* <.001). There was no significant difference in PF of the MTP joint between younger (age < 10: 61.3° ±3.3°) and older subjects (age ≥ 10: 62.5° ±5.5°; U = 22720.00, Z= -1.99, *p* =.05).

## Discussion

Studies investigating the physical profile of ballet and dance students are rare and mostly limited to the female sex, specific age groups and characteristics [15, 16, 18, 22–26]. The present study surveyed physiological characteristics of the lower extremities in female and male students of a vocational ballet institution in Germany.

### Legs and hips

In the present study, all participants showed at least full extension of the knee joints and almost all showed a straight leg axis. Genua recurvata, moreover, were not rare (15%). These results emphasize the leg line as an important aesthetic characteristic of professional classical ballet, which finds underlining in Genua recurvata [6, 27]. Those showed a higher prevalence among the older students in the present study. Particularly in pronounced forms (> 15°), hyperextension should be counteracted by strengthening the ischiocrural musculature, otherwise a subsequent loss of stability leads to an increased risk of acute injuries and overuse injuries [6, 13].

Even more characteristic of classical ballet is the outward rotation of the legs from the hips. A reduced IR in favor of an enlarged ER is typical here [14, 27]. A severely reduced IR, however can also be a sign of hip disease [28]. In Germany, sonographic screening for hip dysplasia is now standard procedure in infancy, which not all of those examined in the present study will have undergone (cross-cultural diversity) [28]. In the present study, the average IR was within the normal range of the adult (30–40°) from the age of 15, and bigger before [29]. While a past study [30] found an extremely high prevalence of (borderline) hip dysplasia in a professional ballet company (89%), the present study documented a severely reduced IR, with a documented suspected diagnosis of hip dysplasia, epiphysiolysis capitis femoris or femoroacetabular impingement, for only 2.2% of those examined. The average ER of examinees (60°) was well above normal values of 30–40° [29], and even 10 degrees greater than ER of ballet students in a study of Hamilton et al. [17], who showed 50°.

The ER is primarily determined by bony factors such as femoral anteversion, the shape of the femoral neck and acetabulum, and to a lesser extent by muscular and ligamentous factors (externally rotatory hip muscles, iliofemoral ligament) [6, 13]. Among bony factors the angle of antetorsion (AT angle) is a major determinant. The smaller it is, the larger the ER [6]. In the general population, the AT angle after completion of growth is about twelve degrees [28]. In dancers, Goertzen et al. [31] found a significantly smaller AT angle (f: 7.6°, m: 10.5°). The AT angle is physiologically still large in infants and decreases until the completion of growth [13].

Exner-Grave [6] describes an explicit detorsion thrust of the femur between the ages of 12 and 14. For a very similar period, Theintz et al. [32] describe the main pubertal bone building processes of the femoral neck (increase in Bone Mass Density). After reaching peak bone mass, remodeling processes lead to a decrease in bone mass density (f/m: from age 14/16) [32]. Compared to the general population, ballet students have environmental factors (lower body weight, shorter body length, higher calcium intake, pubertal developmental retardation, high-performance sport) which could lead to strong interindividual variances [17, 32, 33]. This makes it conceivable that there will be a later peak bone mass attainment with prolonged phase of skeletal malleability among ballet students [34].

Longitudinal studies on vocational ballet students, to examine the bone formation and remodeling processes of the femoral neck, with their effects on the AT angle, are necessary to make more precise statements about the influence of training time and training intensity. The present study found a moderate, negative correlation between age and IR, but only a negligible (positive) correlation between age and ER. Therefore, the extremely high values for ER among examinees in the present study, suggest selection to be an important factor.

The average TT of the present study (22°) was above the average of normal values (15–20°) [6]. None of the students had values (> 40°) associated with an increased risk of damage to the knee joint (high shear forces) in any of the subjects [6]. TT and ER add up to turnout. In the present study, younger students showed a significantly smaller turnout than older students (mean diff.: >10° per leg).

This difference between the age groups is important. Should the turnout be forced, because it is anatomically too small, a ventrally tilted pelvis and a thus provoked hyperlordosis of the lumbar spine, a forced rotation of the knee joints, a hyperpronation of the feet with valgus of the medial malleoli and lateral edges of the feet that lose contact with the ground (rolling-in) become clinically apparent [6, 31]. Such mechanisms of compensation may achieve an average of an additional 25° turnout, but at the same time increase the risk of acute injuries and laxity of the joint-stabilizing ligaments, with consequent degenerative cartilage damage [31, 35, 36, 37, 38]. Though a systematic review in 2020 emphasized the poor quality of existing research and the need for longitudinal studies to verify the causarive relationship between forced turnout and musculoskeletal injuries [39].

Huwyler [13] emphasizes that, to calculate individual turnout, the anatomical ability of the knees for external rotation must not be included. Examinees in the present study showed, on average, a large passive turnout (83° per leg). In comparison, subjects from Hamilton et al. [17] had turnout of 136° (both legs). ROM, however was actively measured here. Thus, the theoretical ideal of a turnout of 180° (90° per leg) was not achieved on average in either study. At this stage, it is unclear what turnout is sufficient in the practical everyday life of classical ballet (although < 180°) without being so small that damaging compensatory mechanisms are resorted to. Gorwa and colleagues [40] could show that students with a passive external hip rotation smaller than 45.8° were likely to engage compensatory mechanisms to achieve full turnout for classical ballet. Though, their study did not assess TT as an important parameter of individual total turnout.

### Foot and ankle complex

The pointed foot completes the leg line in classical ballet. Here in particular, a large PF of the ankle contributes to an aesthetic effect [14, 27]. The present study was able to demonstrate large ballet specific values for PF among participants. They showed significantly higher values, compared to normal (40–50°) [29]. Older students had significantly greater PF than younger students, when correlation with age was negligible (positive). Therefore, longitudinal research is needed to study the possible impact of age, years of training and training intensity on PF. Again selection may play a major role in the high values [13, 14].

In addition to a large PF of the ankle, a high instep contributes to a beautiful foot in classical ballet. In the present study, the prevalence for a high and low instep was the same, which may indicate that instep is not a main selection criterion. Huwyler [13] emphasizes that a hollow foot should not be confused with a high instep. This should always be clarified neurologically and is not suitable for professional ballet [13]. It has been described that repetitive, maximally forced DF of the ankle can lead to symptoms of chronic anterior impingement in dancers and that the anatomy of a pes cavus in particular provokes anterior impingement of the ankle [41–43]. Exner-Grave [6] mitigates that a pes cavus with flexible tarsus and unrestricted DF of the ankle is suitable for professional classical ballet. Accordingly, good joint guidance of the ankle with unrestricted joint play is essential for classical ballet to sufficiently cushion the myriad of repetitive, aestheticized knee bends (i.e. pliés, fondus), small, medium and big jumps. In the present study, average passive DF was within the normal range of the general population (20–30°) [29]. It was observed that dancers in a muscularly warmed-up state can achieve significantly higher values [13]. Participants in the present study were not always muscularly warmed up.

With DF in the ankle, force is optimally developed and transmitted via straight RFA. Valgic or varus RFA in dance, on the other hand, are associated with overuse injuries and injuries at the foot and ankle, and also to the cranial chain at the lower leg, knee, hip and back [28, 36]. The majority of those examined in the present study showed a straight RFA. Valgus RFA, in combination with a flat foot (pes planovalgus), is characteristic of childhood and, moreover, is a very common deformity in adults [6, 28]. Both are also shown by the results of the present study. The varus RFA, which was found more common in older examinees in the present study, could be training-associated, due to exaggerated straightening of the inner legs (overcorrected rolling in).

The development of splayfoot in classical ballet is favored by the repetitive toe stance (relevé) and is not prevented by the material properties of the ballet shoes [4, 6, 35]. This may explain the high prevalence of splayfoot in the present study. Splayfoot, in turn, is associated with the development of Hallux valgus, which is considered to be the most common deformity of the lower extremity [28]. The prevalence of Hallux valgus among the 18 to 65-year-old is reported in the literature to be 23% and positively correlated with age [44]. Among young, female amateur dancers (age 8–16) Hallux valgus was reported with a prevalence of 40,0% [45]. Ishihara and colleagues [46] suggest forced turnout as a relevant risk factor for the development of Hallux valgus. In the present study, prevalence of a valgus axis of the big toe was much lower (16,7%).

A straight axis of the big toe and a large DF in the MTP joint of the big toe guarantee the correct execution of the relevé and the elevation to the tip of the toe. In ballet, the MTP joint of the big toe is as important as the major joints of the body [13]. Compared with normal values for DF (70°), examinees of the present study showed much greater values (89°) [47]. The correlation between age and DF was negligible (positive). Huwyler [48] emphasizes that it cannot be increased by training. Future longitudinal studies are needed to prove such a statement.

The foot can be divided into four categories according to its shape. Regarding work in point shoes, a study could show that, in comparison to the Roman shape, the Egyptian foot is associated with higher instability [49]. Exner-Grave [6] claims that a Roman or Egyptian shape with short toes of equal length provide an even load distribution on toes tips in the female dancer’s pointe shoe. Huwyler [13] claims in a case study that its shape is of medical importance for both sexes. The Greek shape, with an elongated second metatarsal bone, could cause a severely painful clinical picture of the metatarsus [13]. However, in their study, Davidson et al. [50] could not show an increased risk for stress fracture due to a Greek shape of the foot. In the present study, a Greek shape with elongated os metatarsale II was observed in many of the subjects. In contrast, a Roman shape was rather rare in examinees. There is still a lack of studies that investigate complaints of the foot based on foot shape in ballet.

### Limitations

The cross-sectional data collected over the long period of twenty years can be seen as a limitation of the present study. Longitudinal changes and developments over that period of time won’t be represented by this study. Nontheless, this data collection delivers a large data pool on a highly specific population of students at a vocational ballet institution. A population for which not much data is available in current literature.

Furthermore, this study measured passive ROM only. It does not display the possible difference to active ROM. We think that passive ROM represents maximum active possibility. Further research is necessary to identify in which way passive ROM changes (i.e. with age) and if it can be enhanced by years of training or training intensity.

Another limitation is the large age span (5–22 years). As students of the vocational ballet institution, all examinees belong to a very ballet-specific population. Even though vocational training may only start around the age of ten, courses at the vocational institution start from the age of seven. Five students were younger than seven years old (age 5/6: *n* = 2/3) and had been admitted to the courses. Therefore their data is part of this study. We found the data of all students, including those younger than ten, to be interesting information to be published for dance science.

Furthermore, data was not documented for each item and student. We assessed every documented data. Missing data in each characteristic category resulted in different “n”s. In retrospect, it remained unclear why every student was not screened for every characteristic.

### Practical and clinical applications and implications

The present study could show that students of a vocational ballet institution often reach ideal values of the adult professional dancer. However, it could also show that not every student showed ideal values and that differences to ideal values were more evident by age groups. In order to protect the growing body, knowledge of and awareness of those differences are important for teachers and students alike. The present study is able to deliver a data pool on this specific population. It could not answer the following questions: Is there an impact of age, training time and training intensity? Or is ballet-specific selection the only relevant factor for large hip ER, ankle PF and MTP joint DF of the big toe? Longitudinal studies on vocational ballet students are needed in order to better distinguish between the role of age, training time and intensity or natural selection in the process of vocational ballet education.

## Conclusion

The typical examinee of the present study showed a large range of motion for hip ER, ankle PF and MTP joint DF of the big toe. Students were more flexible than examinees of the normal population or ballet students of previous studies. They often achieved ballet-specific ideal values. Differences from the ballet-specific ideal are nonetheless evident and become more clear when looking at different age groups. Awareness for those differences are important for teachers and students to protect the growing artist and athlete from acute injury and chronic overuse damage. The results of the present study do not suggest a possible correlation between age and hip ER, ankle PF or MTP joint DF of the big toe. Hence, it is very likely that a ballet-specific selection favors the generally large joint range of motions found in the present study. The cross-sectional data collected over the long period of twenty years can be seen as a limitation of the present study. Longitudinal data is needed to draw reliable conclusions on developments due to age and training.

## Data Availability

The datasets used and analysed during this study are available from the cor responding author on reasonable request.

## Abbreviations

AT: Antetorsion
DF: Dorsiflexion
ER: External rotation of the hip
IR: Internal rotation of the hip
MTP: Metatarsophalangeal
PF: Plantarflexion
RFA: Rearfoot axis
ROM: Range of Motion
TT: Tibital torsion
